# Sleep Quality, Pain, Worry, and Rumination in Fibromyalgia: Results from Mediation Analyses

**DOI:** 10.3390/jcm14207267

**Published:** 2025-10-15

**Authors:** Michael Tenti, William Raffaeli, Corrado Fagnani, Emanuela Medda, Martina Basciu, Valentina Benassi, Noemi Boschetti, Lorelay Martorana, Sara Palmieri, Giorgia Panini, Leandra Scovotto, Virgilia Toccaceli

**Affiliations:** 1 ISAL Foundation, Institute for Research on Pain, 47921 Rimini, Italy; william.raffaeli@fondazioneisal.it; 2 Cognitive Psychotherapy School and Research Center, Studi Cognitivi, 20121 Milan, Italy; m.basciu_02733_sc@studicognitivi.net (M.B.); v.benassi@allievi.studicognitivi.net (V.B.); n.boschetti@allievi.studicognitivi.net (N.B.); l.martorana@allievi.studicognitivi.net (L.M.); s.palmieri@milano-sfu.it (S.P.); g.panini_03323_sc@studicognitivi.net (G.P.); l.scovotto@allievi.studicognitivi.net (L.S.); 3 Centre of Reference for Behavioral Sciences and Mental Health, Istituto Superiore di Sanità (Italian National Institute of Health), 00161 Rome, Italy; corrado.fagnani@iss.it (C.F.); emanuela.medda@iss.it (E.M.); virgilia.toccaceli@iss.it (V.T.); 4 Department of Psychology, Sigmund Freud University, 20143 Milan, Italy

**Keywords:** fibromyalgia, pain intensity, sleep quality, worry, rumination, repetitive negative thinking, mediation

## Abstract

**Background/Objectives**: Fibromyalgia (FM) is a chronic pain syndrome frequently associated with severe pain, sleep disturbances, worry, and depressive rumination. Although previous studies have shown links among these factors, no study has specifically examined the mediating role of sleep disturbances in the relationship between forms of Repetitive Negative Thinking (i.e., worry and rumination) and pain intensity. This study aimed to investigate whether sleep disturbances mediate the relationship between: (1) worry and pain intensity and (2) depressive rumination and pain intensity. **Methods**: An online cross-sectional survey was conducted with a sample of 867 Italian adults who reported having received an FM diagnosis from a rheumatologist or pain physician. After screening, 733 participants (97.3% female; mean age = 51.0 ± 9.95 years) were included in the analyses. Participants completed the Penn State Worry Questionnaire, the Ruminative Response Scale, the Brief Pain Inventory, and the Pittsburgh Sleep Quality Index. Mediation analyses were performed using Hayes’ PROCESS macro (Model 4). **Results**: Depressive rumination was associated with pain intensity both directly (B = 0.021, 95% Confidence Intervals [CIs] 0.012, 0.030) and indirectly through sleep disturbances (B = 0.014, 95% CIs 0.010, 0.020), indicating partial mediation. In contrast, worry showed no direct effect on pain intensity (B = 0.011, 95% CIs −0.003, 0.025) but demonstrated a significant indirect effect via sleep disturbances (B = 0.018, 95% CIs 0.012, 0.025), consistent with full mediation. **Conclusions**: Pain intensity, sleep quality, worry, and depressive rumination are interrelated in FM. Depressive rumination plays a particularly strong role in pain perception, independent of sleep quality. Interventions that integrate cognitive–behavioral and metacognitive strategies with sleep-focused treatments may help improve both sleep and pain outcomes in individuals with FM.

## 1. Introduction

Chronic pain is a major public health concern in Western countries, with point prevalence estimates ranging from 12% to 48% of the adult population [1,2]. It encompasses a wide range of clinical conditions, including migraine, vulvodynia, low back pain, and other chronic pain syndromes [3,4]. Chronic pain imposes a substantial burden on affected individuals and is associated with high healthcare utilization and indirect costs, such as work disability and reduced quality of life [5].

Fibromyalgia (FM) is among the most disabling chronic pain syndromes [6]. It affects between 0.2% and 6.6% of the general population, predominantly women, with prevalence increasing with age [7,8]. Its clinical impact is amplified by diagnostic challenges, limited treatment options, and frequent social invalidation [9,10]. According to the 2016 American College of Rheumatology criteria [11], FM is primarily characterized by widespread pain lasting more than three months, often accompanied by other symptoms such as fatigue, cognitive difficulties, and depression, which further complicate the clinical picture [9].

Beyond persistent pain, one of the most distressing and distinctive symptoms of FM is sleep disturbance [12,13,14]. Compared to healthy controls, individuals with FM report poorer sleep quality and efficiency, reflected in longer wake times after sleep onset, shorter sleep duration, and lighter sleep in objective assessments, as well as greater difficulties initiating sleep in subjective reports [13]. In a longitudinal study, Bigatti et al. [14] found that baseline sleep disturbances predicted pain one year later, suggesting that sleep plays a pivotal role in the exacerbation of FM symptoms. Evidence also points to sleep dysfunction as a potential pathogenic factor in FM. Experimental studies have shown that sleep deprivation can induce FM-like symptoms in healthy individuals and impair descending pain-inhibitory pathways. Moreover, clinical trials have demonstrated that improving sleep quality leads to reductions in pain [15]. These findings highlight the importance of identifying sleep disturbances and the factors that contribute to poor sleep in individuals with FM, to guide targeted interventions aimed at improving sleep quality.

According to Harvey’s Cognitive Model of Insomnia [16], excessive and negatively valanced cognitive activity can heighten pre-sleep arousal and increase selective attention to internal threat cues (e.g., somatic tension, intrusive thoughts), thereby delaying sleep onset. This delay reinforces the perception of poor sleep quality, which in turn represents a stressor maintaining the cycle of disturbed sleep. Two common forms of heightened cognitive activity are worry and rumination. Worry refers to a chain of uncontrollable thoughts, images, and doubts concerning potential future threats [17]. Rumination, on the other hand, involves a passive and repetitive focus on the causes, implications, and consequences of past stressful events and negative emotions, rather than on their solutions [18]. Worry and rumination are commonly conceptualized as forms of Repetitive Negative Thinking (RNT) [19,20]. Both processes have been observed across a range of psychological and physical health conditions [21,22,23,24,25,26,27].

A recent meta-analysis revealed a consistent association between higher RNT (i.e., worry and rumination) and poorer sleep quality in non-clinical populations [28]. In the context of chronic pain, however, relatively few studies have examined the role of worry and rumination in sleep disturbances. In a sample of patients with benign chronic pain, Smith et al. [29] reported that pre-sleep pain-related thoughts were significantly associated with sleep continuity, independent of depression and nightly pain severity. In a study of adolescents with chronic pain, Palermo et al. [30] found that bedtime worry significantly predicted self-reported sleep quality. Moreover, in a sample of patients with myofascial temporomandibular disorder, Buenaver et al. [31] observed that the rumination component of catastrophizing (i.e., worrying about pain with an inability to shift attention away from pain-related thoughts [32]) had a significant indirect effect on pain intensity through sleep disturbance. This effect was not observed for the helplessness or magnification components of catastrophizing. Qualitative evidence further supports these findings. In a study by Edwards et al. [33], over one-quarter of participants spontaneously reported experiencing nocturnal rumination. Many described being unable to “switch off” when trying to fall asleep, with some even ruminating about their inability to sleep. Notably, approximately 30% of participants indicated that rumination negatively impacted their sleep, primarily by delaying sleep onset.

Taken together, these findings underscore the role of RNT processes, such as worry and rumination, in disrupting sleep among individuals with chronic pain. Importantly, some evidence also suggests that worry and rumination may exacerbate pain itself, contributing to greater pain intensity by heightening attentional focus on pain-related cues and sustaining negative affective states [34,35,36,37,38]. However, other studies have reported no significant correlation between pain intensity and worry or rumination [39,40]. These inconsistencies may reflect methodological differences, such as variability in sample characteristics or measurement instruments.

Understanding these relationships is critical for identifying modifiable targets for interventions aimed at reducing both pain and sleep disturbances in individuals with FM. Despite growing research on the complex interplay between pain intensity, sleep quality, worry, and rumination, a critical gap remains: to date, no study has systematically examined whether sleep quality mediates the associations between these distinct RNT processes and pain intensity.

The present study aimed to address this gap by investigating two separate mediation models in a large sample of individuals with FM. Specifically, we examined whether sleep quality mediate the relationship between: (1) worry and pain intensity, and (2) depressive rumination and pain intensity. We hypothesized that both worry and depressive rumination would be indirectly associated with higher pain intensity through increased sleep disturbances.

## 2. Materials and Methods

This cross-sectional study involved a convenient sample of adults with FM (*n* = 867) recruited online in March 2024 through the Facebook Page of “Comitato Fibromialgici Uniti-Italia Odv”, an Italian association of FM patients.

To be eligible, participants had to be aged 18 years or older and report having received a diagnosis of FM from a rheumatologist or pain physician.

The Ethics Committee of the Sigmund Freud University (Milan, Italy) approved the study (Ref. ID22N2WHC11M1V90684, 20 February 2024). Study objectives and procedures were explained to participants, who provided informed consent before starting the online survey. All procedures were conducted in accordance with the Declaration of Helsinki (1964, as revised in October 2024).

### 2.1. Measures

The online survey collected information on socio-demographic characteristics, FM diagnosis, time lag between symptoms onset and FM diagnosis, pain duration, and perceived effectiveness of current treatments. Treatment effectiveness was assessed using a single 11-point Numerical Rating Scale: “If you take medications or undergo therapies for pain, how effective do you consider them?”. Responses ranged from 0 (not effective at all) to 10 (completely effective).

Worry was measured using the Italian version of the Penn State Worry Questionnaire (PSWQ) [41]. The PSWQ is a 16-item self-report measure that assesses the general tendency to worry, focusing on its pervasiveness, persistence, and uncontrollability, rather than on the specific content of anxious thoughts (e.g., Item 7: “I’m always worrying about something”). Items are rated on a 5-point Likert scale ranging from 1 (“Not at all typical of me”) to 5 (“Very typical of me”). Total scores range from 16 to 80, with higher scores indicating greater levels of worry. In the present study, the PSWQ demonstrated excellent internal consistency (α = 0.92).

Depressive rumination was assessed using the Italian version of the Ruminative Response Scale (RRS) [42]. This 22-item self-report questionnaire, based on the Response Style Theory, conceptualizes rumination as a passive and repetitive focus on depressive symptoms, their causes, and consequences. Items are rated on a 4-point Likert scale ranging from 1 (almost never) to 4 (almost always). Total scores range from 22 to 88, with higher scores indicating greater levels of depressive rumination. In the present study, the RRS showed excellent internal consistency (α = 0.94).

Pain intensity was measured using the pain intensity subscale of the Italian version of the Brief Pain Inventory (BPI) [43], a widely used multidimensional instrument for assessing pain. The subscale includes four items: (1) worst pain in the past 24 h, (2) least pain in the past 24 h, (3) average pain in the past 24 h, and (4) current pain. Each item is rated on a numerical scale from 0 (no pain) to 10 (pain as bad as you can imagine). Higher scores indicate greater pain intensity. The total pain intensity score was calculated by averaging the four items, yielding a score from 0 to 10. In the present study, internal consistency for the pain intensity subscale was good (α = 0.87).

Sleep quality were assessed using the Italian version of the Pittsburgh Sleep Quality index (PSQI) [44]. The PSQI consists of 19 items and five additional questions used for clinical purposes. The questionnaire evaluates various aspects of sleep, including duration, latency, frequency, and severity of sleep problems. Each item contributes to one of seven subscales, scored from 0 to 3, with a global score ranging from 0 to 21. Higher scores indicate poorer sleep quality (to improve clarity in the Results section, we refer to higher PSQI scores as “sleep disturbances”, emphasizing that higher values reflect worse sleep quality). In the internal consistency analysis, the subscale “Use of sleep medication” showed a low corrected item–total correlation (0.104). Including this subscale yielded a Cronbach’s alpha of 0.62, whereas removing it, alpha increased to 0.70, indicating acceptable internal consistency for the remaining subscales. Consequently, the subscale was excluded from the calculation of the total PSQI score to enhance reliability. Similar issues with this subscale have been reported in previous studies, suggesting that it often performs differently from the other PSQI components across various populations [45,46].

### 2.2. Data Analysis

Descriptive statistics were used to summarize participants’ demographic and pain-related characteristics. Missing data were first examined by calculating the percentage of missing responses for each item. When the percentage of missing values was below 10%, missing data were imputed using the item mean. Cases with more than 10% missing data were excluded through listwise deletion [47]. Preliminary checks for normality were performed by inspecting skewness and kurtosis, with acceptable values ranging between −1 and +1. Pearson’s correlations were calculated among the study variables. Associations between pain intensity (outcome) and potential covariates (e.g., age, perceived treatment efficacy) were also examined. For variables with non-normal distributions, Spearman’s rho was used instead. For the interpretation of correlation magnitudes, we followed the guidelines of Gignac and Szodorai [48], who recommend considering correlations of 0.10, 0.20, and 0.30 as relatively small, typical, and relatively large, respectively. Variables showing a significant correlation with either the mediators or the outcome (r ≥ 0.30) were included as covariates [49]. Multivariate outliers were identified using Mahalanobis distance, applying a chi-square cut-off of *p* < 0.001 [50]. Multicollinearity was assessed through the Variance Inflation Factor (VIF), with values below 5 considered acceptable [51].

Mediation analyses were performed in IBM SPSS Statistics version 25.0 (IBM Corp., Armonk, NY, USA) using the PROCESS macro (model 4; Version 4.1). Two mediation models were tested to examine whether sleep quality mediated the relationships between (1) worry and pain intensity, and between (2) depressive rumination and pain intensity. Indirect effects were estimated using bootstrapping with 5000 resamples. A 95% confidence intervals (CIs) that did not include zero was considered as evidence of a statistically significant indirect effect [52]. All other analyses were performed in IBM SPSS Statistics version 25.0 (IBM Corp., Armonk, NY, USA). All statistical tests were two-tailed and results were considered significant at *p* < 0.05.

## 3. Results

Of the 867 questionnaires initially collected, 89 were excluded due to more than 10% missing responses on PSQI, resulting in a valid response rate of 89.7%. A comparison between respondents and non-respondents revealed no significant differences in age, pain duration, perceived treatment effectiveness, or worry. However, significant differences emerged in pain intensity (F = 14.614, *p* < 0.001) and depressive rumination (F = 7.603, *p* < 0.01).

Among the 778 participants with valid responses, 734 (94.3%) reported having received a FM diagnosis from either a rheumatologist or a pain physician and were therefore considered eligible for inclusion in the study. The final sample thus comprised 734 participants.

Table 1 summarizes the sociodemographic and pain-related characteristics of the final sample.

### 3.1. Preliminary Analysis

A multivariate outlier was identified and removed from the database, resulting in a final working sample of 733 participants. The ranges of skewness (−0.44 to 0.20) and kurtosis (−0.60 to 0.30) for all study variables supported the assumption of normality, except for pain duration, which showed a non-normal distribution (skewness = 1.332; kurtosis = 1.854). VIF values (1.077–1.56) were below the recommended threshold, indicating no issues with multicollinearity.

Table 2 presents descriptive statistics and intercorrelations among the study variables. In the correlation analyses, pain intensity showed a relatively small positive correlation with worry (r = 0.142), a relatively typical correlation with depressive rumination (r = 0.279), and a relatively large correlation with sleep disturbances (r = 0.363). Among the cognitive and sleep variables, worry and rumination were strongly correlated (r = 0.600, well above the large threshold), worry and sleep disturbances showed a relatively typical association (r = 0.250), and depressive rumination and sleep disturbances showed a moderately large correlation (r = 0.380). These findings indicate that all variables are positively related, with the strongest links observed between worry and depressive rumination and between depressive rumination and sleep disturbances, highlighting the close interplay among these cognitive and sleep-related processes. Regarding potential covariates: age was negatively correlated with rumination (r = −0.081, *p* < 0.01; small correlation); perceived treatment effectiveness was negatively correlated with pain intensity (r = −0.195, *p* < 0.001; small-to-typical correlation), sleep disturbances (r = −0.191, *p* < 0.001; small-to-typical correlation), worry (r = −0.155, *p* < 0.001; small correlation), and rumination (r = −0.104, *p* < 0.001; small correlation); pain duration was positively correlated with sleep disturbances (rho = 0.101, *p* < 0.01; small correlation). All correlation coefficients were below the cut-off of r = 0.30; therefore, no covariates were included in the mediation models.

### 3.2. Testing Mediation Models

#### 3.2.1. Simple Mediation with Pain Intensity as the Outcome and Depressive Rumination as the Predictor

**Figure 1:**
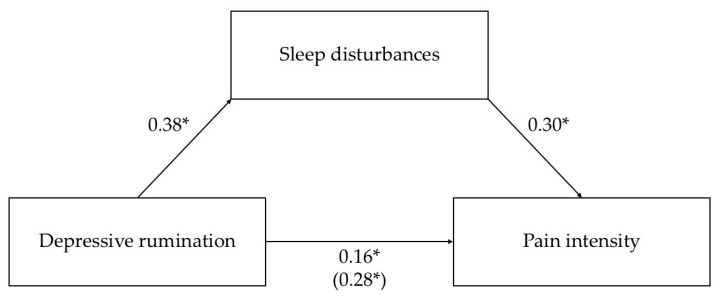
Mediation model with depressive rumination as the predictor of pain intensity through sleep disturbances. Standardized parameter estimates are shown, and the beta for the total effect is reported in parentheses. Solid lines indicate significant paths; dashed lines indicate non-significant paths. * *p* < 0.001.

In the mediation analysis with depressive rumination as the predictor, higher levels of depressive rumination were directly associated with greater pain intensity. Regarding the indirect pathway, depressive rumination also exerted a significant indirect effect on pain intensity via sleep disturbances. Specifically, higher depressive rumination was linked to more severe sleep disturbances, which, in turn, were associated with higher pain intensity. These findings indicate a partial mediation, suggesting that while depressive rumination directly contributes to pain perception, part of its effect operates through sleep disturbances. Overall, the model explained 8% (R^2^ = 0.08) of the variance in pain intensity. The detailed regression-based mediation results are reported in Table 3. The total, direct, and indirect effects are presented in Table 4. Standardized path coefficients are shown in Figure 1.

#### 3.2.2. Simple Mediation with Pain Intensity as the Outcome and Worry as the Predictor

**Figure 2:**
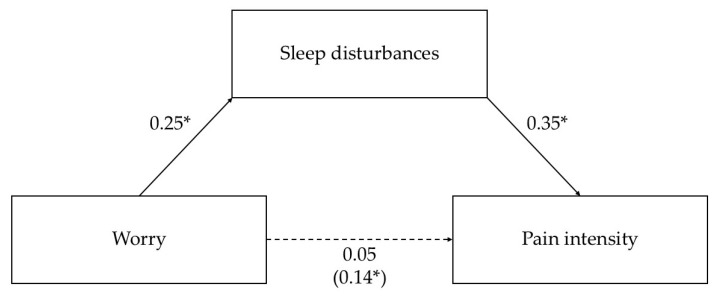
Mediation model with worry as the predictor of pain intensity through sleep disturbances. Standardized parameter estimates are shown, and the beta for the total effect is reported in parentheses. Solid lines indicate significant paths; dashed lines indicate nonsignificant paths. * *p* < 0.001.

In the mediation analysis with worry as the predictor, the direct effect of worry on pain intensity was non-significant. However, worry showed a significant indirect effect on pain intensity via sleep disturbances, indicating full mediation. Specifically, higher levels of worry were associated with more severe sleep disturbances, which, in turn, were linked to higher pain intensity. These results suggest that sleep disturbances fully account for the relationship between worry and pain intensity in this sample. Overall, the model explained 2% (R^2^ = 0.02) of the variance in pain intensity. The corresponding regression-based mediation results are reported in Table 5. The total, direct, and indirect effects are shown in Table 6. Standardized path coefficients are shown in Figure 2.

## 4. Discussion

Despite growing evidence on the relationship between pain intensity, sleep quality, worry, and rumination, no study to date has examined whether sleep quality mediates the associations between these RNT processes and pain intensity.

Our findings support Harvey’s Cognitive Model of Insomnia [16], highlighting a pathway in which worry and depressive rumination contribute to increased sleep disturbances. This is consistent with previous research showing that RNT processes interfere with sleep continuity and quality in both clinical and non-clinical populations [28,29,30,31,33]. By demonstrating that these mechanisms also operate in FM, our results extend previous work and underscore the role of maladaptive cognitive activity in perpetuating poor sleep quality in this population.

Furthermore, our results underscore the negative impact of sleep disturbances on pain intensity. This finding aligns with a substantial body of evidence indicating that sleep disturbances are a robust predictor of pain exacerbation across various chronic pain conditions, including FM. Notably, longitudinal and micro-longitudinal studies have shown that sleep impairments more consistently predict subsequent pain than the reverse, supporting the primacy of sleep dysfunction in the pain–sleep relationship [53]. Several mechanisms may explain why poor sleep quality has such a strong effect on pain. Mediation studies have highlighted the roles of depression, anxiety, attention to pain, pain helplessness, stress, fatigue, and reduced physical activity as important factors linking sleep disturbances to greater pain intensity [54]. At the neurobiological level, preliminary evidence suggests that alterations in dopaminergic and opioidergic systems may contribute to this relationship. However, these mechanisms remain incompletely understood and require further investigation [53].

Another important finding of the present study concerns the direct effect of depressive rumination on pain intensity, independent of sleep disturbances. This suggests that rumination may exacerbate pain not only through its disruptive effects on sleep but also by maintaining negative affective states and attentional biases toward pain-related cues, thereby amplifying the subjective experience of pain. This interpretation is consistent with experimental and neuroimaging evidence indicating that rumination intensifies attentional and anticipatory processes related to pain. For instance, Brookes et al. [55] demonstrated that experimentally induced rumination increased both distress and pain perception during an acute pain task, partly by promoting a vigilance-avoidance attentional pattern toward pain-related stimuli. Similarly, Kokonyei et al. [56] found that trait rumination was associated with heightened neural responses during both pain anticipation and perception, as well as with impaired detection of unexpected relief, suggesting that rumination may intensify pain through altered cognitive and emotional processing. Collectively, these findings reinforce the notion that depressive rumination is a cognitive factor that directly exacerbates pain perception, highlighting its relevance as a potential target for psychological interventions in chronic pain management.

From a clinical perspective, these findings highlight the importance of interventions specifically aimed at reducing RNT, which appear to play a role in disrupting sleep and amplifying pain in FM. Metacognitive Therapy (MCT) [57] and Rumination-Focused Cognitive–Behavioral Therapy (RF-CBT) [20] represent promising approaches. Specifically, MCT focuses on identifying and modifying dysfunctional metacognitive beliefs (e.g., “My worrying is uncontrollable”, “Worrying helps me to cope…”) that perpetuate worry and rumination. In doing so, it helps individuals disengage attention from intrusive thoughts and RNT, rather than trying to challenge or suppress their content. MCT has demonstrated efficacy in treating a range of emotional disorders, including depressive disorders, generalized anxiety disorder, social anxiety disorder, and other conditions characterized by excessive worry and rumination [58,59,60,61]. These findings suggest that MCT could be adapted to address similar cognitive processes in FM, although it has not yet been tested in this specific population.

Similarly, RF-CBT specifically addresses depressive rumination by helping individuals recognize and modify unhelpful thinking styles, such as abstract, repetitive, and passive thought patterns, and replace them with more concrete and constructive coping strategies. This intervention has been shown to reduce depressive rumination and improve emotional functioning in individuals with depression [20]. However, to date, no randomized controlled trials have tested this intervention in patients with FM or chronic pain.

Although these approaches have not yet been tested in FM or chronic pain populations, their theoretical framework directly target the cognitive mechanisms identified in this study. Integrating them into multimodal pain management programs could potentially improve outcomes by breaking the cycle of negative thinking, poor sleep, and heightened pain perception.

In addition, mindfulness-based interventions have demonstrated positive effects on both sleep quality and emotional regulation in chronic pain populations, including FM. A recent study showed that a mindfulness-based stress reduction program improved sleep quality over time [62]. This benefit may result from reducing physiological and cognitive arousal while promoting present-moment awareness and non-judgmental acceptance, thereby counteracting rumination and worry that frequently disrupt sleep.

Future clinical trials are needed to evaluate whether interventions explicitly targeting rumination and worry can improve sleep quality and, consequently, pain outcomes. If confirmed, this would broaden the current range of psychological treatments for chronic pain beyond traditional CBT approaches, opening avenues for tailored interventions addressing transdiagnostic cognitive mechanisms such as RNT.

This study has several limitations. First, the cross-sectional design precludes causality inferences. Future longitudinal studies could provide a dynamic understanding of changes in sleep, cognitive processes, and pain over time, allowing stronger conclusions regarding causal relationships. Second, online data collection through FM-specific patients associations, while cost-effective, may have introduced selection bias and reduced sample representativeness. Individuals without Internet access or with limited digital literacy were likely excluded, and the sample may have been biased toward more engaged participants, limiting generalizability. Third, the lack of control over the testing environment inherent to online surveys may have affected response quality. Future studies could consider diversified recruitment strategies and incorporate attention checks or social desirability measures to improve data quality. Fourth, the internal consistency of the PSQI was lower than typically recommended. In line with prior research [63,64,65,66], one subscale with a particularly low item–total correlation was excluded to improve reliability. While this step strengthened the measure’s internal consistency, it may have reduced comparability with studies using the full scale. Fifth, a preliminary comparison between respondents and non-respondents revealed significant differences in pain intensity and depressive rumination. This suggests a potential response bias, with participants who completed the survey possibly experiencing higher levels of pain and rumination, further limiting generalizability. Sixth, the proposed mediation models accounted for a small proportion of variance in pain intensity. This is not be surprising, since FM pain is a multifaceted phenomenon influenced by numerous biopsychosocial factors [67]. While rumination and worry contribute to sleep disturbances and pain, they are insufficient to fully explain their variability. Other factors, such as physical activity, comorbid mood or anxiety disorders, and pain coping strategies, may also play important roles and should be considered in future research. Moreover, hormonal, neurochemical, and inflammatory changes may further contribute to the complexity of sleep disturbances and pain mechanisms in FM [68,69,70,71,72]. The absence of a healthy control group or other clinical comparison cohorts represents a further limitation of the present study. Therefore, the specificity of these findings to FM cannot be determined, and future studies should include healthy controls and clinical groups with other chronic pain or affective conditions to disentangle the FM-specific versus transdiagnostic nature of these associations. This could be particularly relevant considering that previous research have shown that certain forms of RNT (specifically, worry and anger rumination) may be more pronounced in individuals with FM compared to both healthy controls and patients with other rheumatologic conditions [73]. Moreover, the mean PSQI global score observed in our sample (M = 13.45) was substantially higher than the clinical cutoff of 5 and also exceeded the mean value reported in a comparative study involving six medical populations (M = 8.0) [74]. Finally, since anxiety and depression are common comorbidities in FM and were not controlled in the present analyses. We avoided adding further self-report measures to limit participant burden and maximize data quality, considering the frequent attention and concentration difficulties reported by people with FM. However, future research should include these variables to clarify their potential confounding roles.

In conclusion, our findings extend current knowledge on the interplay among worry, rumination, sleep quality, and pain in FM. They emphasize the importance of targeting worry and rumination in psychological interventions aimed at improving sleep quality and pain intensity. Future research should investigate whether reducing these RNT processes can concurrently improve sleep and pain outcomes. If confirmed, such findings could inform the development of more tailored and mechanistically driven treatments for FM and other chronic pain populations.

## Figures and Tables

**Table 1:** Participants’ socio-demographic and pain characteristics (*n* = 734).

Variable	*n* (%)	Mean (SD), Range
**Gender**		
Male	19 (2.6)	
Female	714 (97.3)	
Other	1 (0.1)	
**Age (years)**		50.92 (9.87), 18–76
**Education**		
Primary to Middle school	138 (18.8)	
High school	386 (52.6)	
University degree	151 (20.6)	
Postgraduate	59 (8)	
**Occupation**		
Unemployed	177 (24.1)	
Employed	474 (64.6)	
Student	5 (0.7)	
Retired	78 (10.6)	
**Housing situation**		
Living alone	94 (12.8)	
Living with parents	47 (6.4)	
Living with current family	582 (79.3)	
Living with other non-family people	11 (1.5)	
**Time since FM diagnosis**		
Less than 1 year	101 (13.8)	
From 1 to 3 years	264 (35.9)	
From 4 to 5 years	101 (13.8)	
From 6 to 10 years	96 (13.1)	
More than 10 years	172 (23.4)	
**Pain duration (years) (*n* = 718)**		13.86 (10.22), 0–55
**Undergoing treatment**		
Yes	574 (78.2)	
No	160 (21.8)	
**Perceived treatment effectiveness (*n* = 620)**		4.34 (2.37), 0–10

**Table 2:** Descriptive statistics and correlations among the study variables (*n* = 733).

		Pearson’s r Correlation			
Questionnaire	Mean (SD)	1	2	3	4
1. Pain intensity	6.08 (1.78)		0.142 *	0.279 *	0.363 *
2. Worry	51.69 (8.75)			0.600 *	0.250 *
3. Depressive rumination	53.14 (14.07)				0.380 *
4. Sleep disturbances	13.45 (3.79)				

* *p* < 0.001.

**Table 3:** Regression-based results of the mediation model with depressive rumination as the predictor of pain intensity.

Criterion	Predictors	R	R2	F	β	t	95% CIs
Sleep disturbances	Depressive rumination	0.38	0.14	123.27	0.10	15.79	[7.011, 9.002]
Pain intensity	Depressive rumination	0.39	0.15	66.83	0.02	4.47	[0.012, 0.030]
	Sleep disturbances				0.14	8.16	[0.107, 0.175]

**Table 4:** Total, direct, and indirect effects in the mediation model with depressive rumination as the predictor of pain intensity.

Effect	B	SE(β)	95% CIs	β	SE(β)	95% CIs
Total effect	0.035	0.005	[0.026, 0.044]	0.28		
Direct effect	0.021	0.005	[0.012, 0.030]	0.16		
Indirect effect	0.014	0.002	[0.010, 0.020]	0.11	0.02	[0.083, 0.149]

**Table 5:** Regression-based results of the mediation model with worry as the predictor of pain intensity.

Criterion	Predictors	R	R2	F	B	t	95% CIs
Sleep disturbances	Worry	0.25	0.06	48.86	0.11	6.99	[0.078, 0.139]
Pain intensity	Worry	0.37	0.13	56.64	0.01	1.52	[−0.003, 0.025]
	Sleep disturbances				0.16	9.82	[0.131, 0.197]

**Table 6:** Total, direct, and indirect effects in the mediation model with worry as the predictor of pain intensity.

Effect	B	SE(B)	95% CIs	β	SE(β)	95% CIs
Total effect	0.029	0.008	[0.014, 0.043]	0.14		
Direct effect	0.011	0.007	[−0.003, 0.025]	0.05		
Indirect effect	0.018	0.003	[0.012, 0.025]	0.09	0.01	[0.060, 0.118]

## Data Availability

The data presented in this study are available on reasonable request from the corresponding author.

## Abbreviations

The following abbreviations are used in this manuscript:

BPI	Brief Pain Inventory
FM	Fibromyalgia
PSQI	Pittsburgh Sleep Quality Index
PSWQ	Penn State Worry Questionnaire
RNT	Repetitive Negative Thinking
RRS	Ruminative Response Scale
SD	Standard Deviation
